# A new combined strategy to implement a community occupational therapy intervention: designing a cluster randomized controlled trial

**DOI:** 10.1186/1471-2318-11-13

**Published:** 2011-03-30

**Authors:** Carola ME Döpp, Maud JL Graff, Steven Teerenstra, Eddy Adang, Ria WG Nijhuis - van der Sanden, Marcel GM OldeRikkert, Myrra JFJ Vernooij-Dassen

**Affiliations:** 1 Scientific Institute for Quality of Healthcare (IQ healthcare), Radboud University, Nijmegen Medical Centre, Nijmegen, the Netherlands; 2 Alzheimer Centre Nijmegen, Radboud University Nijmegen Medical Centre, Nijmegen, the Netherlands; 3 Dept. of Rehabilitation-Occupational Therapy, Radboud University Nijmegen Medical Centre, Nijmegen, the Netherlands; 4 Dept. of Epidemiology, Biostatistics and HTA, Radboud University Nijmegen Medical Centre, Nijmegen, the Netherlands; 5 Dept. of Rehabilitation, Radboud University Nijmegen Medical Centre, Nijmegen, the Netherlands; 6 Department of Geriatrics, Alzheimer Centre Nijmegen, Radboud University Nijmegen Medical Centre, Nijmegen, the Netherlands; 7 Dep. of Primary and Community Care, and Alzheimer Center Nijmegen, Radboud University Nijmegen Medical Centre, Nijmegen, the Netherlands; 8 Kalorama Foundation, Radboud University Nijmegen Medical Centre, Nijmegen, the Netherlands

## Abstract

**Background:**

Even effective interventions for people with dementia and their caregivers require specific implementation efforts. A pilot study showed that the highly effective community occupational therapy in dementia (COTiD) program was not implemented optimally due to various barriers. To decrease these barriers and make implementation of the program more effective a combined implementation (CI) strategy was developed. In our study we will compare the effectiveness of this CI strategy with the usual educational (ED) strategy.

**Methods:**

In this cluster randomized, single-blinded, controlled trial, each cluster consists of at least two occupational therapists, a manager, and a physician working at Dutch healthcare organizations that deliver community occupational therapy. Forty-five clusters, stratified by healthcare setting (nursing home, hospital, mental health service), have been allocated randomly to either the intervention group (CI strategy) or the control group (ED strategy). The study population consists of the professionals included in each cluster and community-dwelling people with dementia and their caregivers. The primary outcome measures are the use of community OT, the adherence of OTs to the COTiD program, and the cost effectiveness of implementing the COTiD program in outpatient care. Secondary outcome measures are patient and caregiver outcomes and knowledge of managers, physicians and OTs about the COTiD program.

**Discussion:**

Implementation research is fairly new in the field of occupational therapy, making this a unique study. This study does not only evaluate the effects of the CI-strategy on professionals, but also the effects of professionals' degree of implementation on client and caregiver outcomes.

**Clinical trials registration:**

NCT01117285

## Background

Dementia is associated with a major decrease in quality of life of clients and their caregivers due to a loss of independence, autonomy, and social participation [1]. In addition, dementia is a major driver of costs in health care [2]. These costs increased by 34% between 2005 and 2009 [3]. In the Netherlands, nearly 1% of people aged 65 years old suffer from dementia and 40% of people aged 90 and over [4]. The number of dementia patients will increase substantially in the years to come [5]. This stresses the importance of effective interventions which aim at increasing quality of life of people with dementia and their caregivers [6] and implementation of these interventions in practice.

Two recent pilot studies showed that strategies currently used to implement the COTiD program are not effective (Graff & Van Uden: Pilot research to the implementation by trained OTs of the COTiD program, unpublished)

(van't Leven, Graff, Kaijen, de Swart, OldeRikkert, Vernooij-Dassen: Implementing of an effective occupational therapy guideline for older persons with dementia and their informal caregivers: facilitating and impeding factors, submitted). It was evaluated if a post-graduate course on working with community occupational therapy in dementia (COTiD) program was sufficient in establishing implementation in practice (Graff & Van Uden, unpublished). Although the COTiD program was proven to be an effective [7] and cost effective intervention [8], only 20% (Graff & Van Uden, unpublished) of the trained OTs used the program completely or partly due to existing barriers (van 't Leven et al, submitted). These findings are in agreement with previous studies reporting on the ineffectiveness of post-graduate courses and workshops with regard to the use of new knowledge in practice [9-11].

To make sure patients with dementia and their caregivers are able to receive and benefit from occupational therapy according to the COTiD program, a combined implementation (CI) strategy was developed addressing the existing barriers to implementation (van 't Leven et al, submitted). A multifaceted strategy was created as previous studies found this to be most effective in changing professional's behavior [9,12,13]. The combined implementation (CI) strategy exists of various strategies intended to improve OTs' adherence to the COTiD program, increase community OT use, and to increase managers' and physicians' knowledge and attitudes regarding the COTiD program.

The current study aims to compare the effectiveness of the CI-strategy with the effectiveness of the usual educational (ED) strategy in increasing both OTs adherence to the COTiD program and the use of community OT. In addition, the cost-effectiveness of the CI-strategy is compared to the cost-effectiveness of the ED-strategy. In this article, the design of this cluster randomized trial is described according to the latest CONSORT guidelines of randomized controlled trials on non-pharmacological interventions [14].

## Methods/Design

### Trial Design

**Figure 1:**
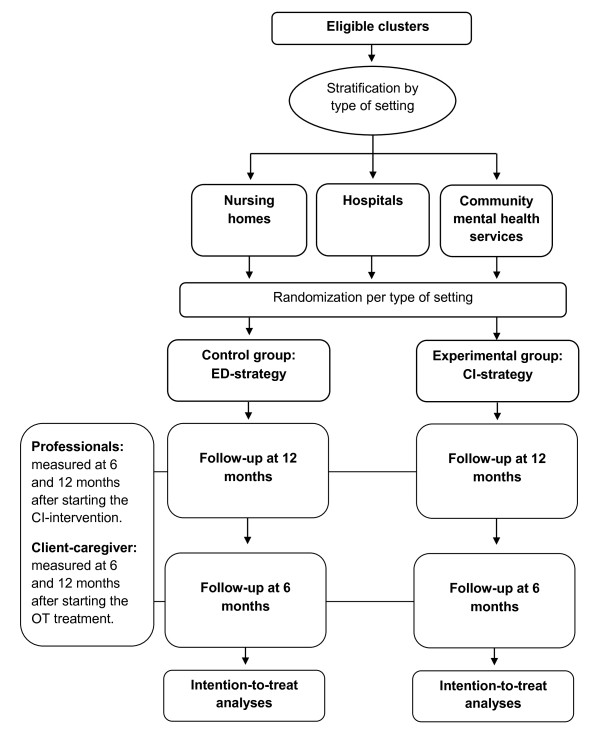
Flow Chart of the Study Design.

A single blinded, cluster randomized controlled design is used to compare the effectiveness and cost-effectiveness of the two implementation strategies. An independent statistician stratified the clusters by type of setting (hospital, nursing home, and mental health services) and randomized them to either the control (educational strategy) or experimental group (the combined implementation strategy) (see Figure 1). Clusters were randomized using a 2 to 1 (control vs. experimental) ratio, as it is expected that physicians in the experimental group will refer more clients for this community occupational therapy intervention than those in the control group. This assumption implies that more clusters are needed in the control group to collect data from a sufficient number of client-caregiver couples. At the time of randomization 45 organizations agreed to participate in the study.

Three research assistants blinded for group allocation collected all data. Client-caregiver couples are not aware of the group allocation of their healthcare professionals (physician and occupational therapist). A complete double blinded trial is not possible as the participating professionals are aware of the implementation strategy they received.

### Participants

#### Healthcare professionals

A cluster consists of occupational therapists, managers, and physicians working at a healthcare setting which delivers community occupational therapy (outpatient service general hospital, outpatient treatment from nursing home & outpatient community mental health services). Clusters were preferably formed out of professionals working at the same organization. However, in several cases professionals from different organizations formed a cluster as these were the usual networks in current clinical practice. Clusters were approached between January and December of 2009 and asked to participate in the study.

Eligibility criteria for clusters:

1. Clusters consist of at least two OTs, one physician, and one manager

2. Outpatient occupational therapy treatment is provided by the cluster

3. Each cluster is able to include at least 8 client-caregiver couples in the study

4. OTs within a cluster completed the post-graduate course on the COTiD program before the start of the study.

#### Client-caregiver couples

Clients with dementia and their caregiver are enrolled during the first year of the study. They are approached to participate in the study by physicians of one of the study clusters.

Eligibility criteria for client-caregiver couples:

1. The client has mild to moderate dementia (MMSE score 10-24, DSM IV criteria for dementia)

2. Clients are referred to an occupational therapist participating in the study

3. The client lives at home at the time of inclusion

4. The informal caregiver takes care of the client at least two times a week

5. The client is not diagnosed with depression (Geriatric Depression Scale 30 >12)

6. The client has no severe behavioral or psychological symptoms (BPSD)

7. The client has no severe illness preventing participation

8. The caregiver has no severe illness preventing participation

9. Both client and caregiver consent to participate

Participation of client-caregiver couples is discontinued when severe BPSD develops, the client is permanently admitted to an institution, or the client or caregiver no longer wish to participate.

### Interventions

#### The Educational Strategy

Occupational therapists, physicians, and managers do not receive any intervention during the study period. Occupational therapists only received the basic three-day post-graduate course before the start of the study. This course provides background and theory on the COTiD program, and is mainly focused on skill training. Skills are trained by role-playing and homework assignments that involve video-taping actual OT sessions. In addition, OTs are asked to complete reading assignment between class meetings. The experimental strategy is offered to the control group after completion of the study.

#### The Combined Implementation Strategy

The CI-strategy is a multifaceted strategy that, beside the post-graduate course for OTs, consists of additional interventions toward occupational therapists and interventions toward physicians and managers:

*1. Implementation training days*. The training days focus on refreshing and/or increasing professional skills regarding execution of the COTiD program and skills regarding promotion of the COTiD program.

*2. Coaching on the job*. Coaching sessions are held to address individual problems experienced by occupational therapists regarding the implementation of the COTiD program. Between five and seven coaching sessions are scheduled depending on the OTs individual needs.

*3. Regional meetings*. These meetings are provided to create an opportunity for OTs to discuss practice issues with OT colleagues from the same region. Four regional meetings are organized during one year.

*4. Web-based reporting system and discussion forum*. An electronic reporting system was developed to guide OTs through the steps of the COTiD program. Treatment reports can be created for every client-caregiver couple. In addition, a link to a discussion platform is provided through which OTs are able to share experiences and exchange helpful resources.

*5. Website and newsletters*. Information on the COTiD program, its effectiveness, and cost-effectiveness is provided to physicians, managers, and OTs. The information is presented through a website and four newsletters. Information is adjusted for each group of professionals, to meet the specific needs of the group.

*6. Telephone calls*. Managers and physicians are contacted by phone at least once to evaluate if they have questions on the COTiD program and the implementation in practice. The goal is to provide more insight in the COTiD program and to motivate managers to facilitate the program and motivate physicians to refer clients to treatment according to the COTiD program. The ultimate goal is to increase the number of referrals to community OT.

Two OTs who are experienced teachers, have extensive experience in using the COTiD program, and are trained in motivational interviewing provide the implementation training, coaching, and organize the regional meetings.

The elements of the CI-strategy are selected to meet the barriers found during the pilot study (van 't Leven et al, submitted). To meet the need for feedback and guidance expressed by occupational therapists in the pilot study (van 't Leven et al., submitted) the two training days and coaching on the job were included. Guidance and structure in using the COTiD program is also offered by providing access to the web-based reporting system. The training days and the regional meetings are opportunities to meet colleagues and create a network, which can be used for guidance and feedback both during and after the intervention.

Both managers and physicians are part of the organizational structure in healthcare. They have an important role in the facilitation of occupational therapy. Managers of occupational therapy services need to facilitate the conditions to be able to offer OT according to the COTiD program. Physicians are needed to get eligible clients referred to occupational therapy services. However, one of the pilot studies found that managers and physicians had a lack of knowledge about the COTiD program or even occupational therapy in general (van 't Leven et al., submitted). Therefore, strategies focused on these professionals such as the educational website, newsletters, and personal phone calls were included in the CI strategy.

The interventions toward managers and physicians have an educational nature. Although educational strategies are only slightly effective in changing behavior [15], acquiring knowledge is essential before making a decision to change behavior or not [16]. Beside the educational interventions managers and physicians are motivated during the phone calls.

### Outcome measures

Data are collected from professionals (OTs, managers, and physicians) and client-caregiver couples. Data from professionals are gathered at baseline (T_0_), 6 months (T_1_), and 12 months (T_2_) (Table 1). Information from client-caregiver couples is collected at baseline (T_0_), 3 months (T_1_), 6 months (T_2_), 9 months (T_3_), and 12 months (T_4_) (Table 2).

**Table 1:** Overview of outcome measures on professional level

Variable	PO	SO	EE	BG	Instrument/Source	T0	T1	T2
Demographics				✓	Survey	✓		
Adherence	✓		✓		Vignettes & CAQ	✓	✓	✓
Use of Community OT	✓							
Strategy costs	✓		✓		Costs registered by SPs Costs survey OTs, MGs, MDs		✓	✓
Referral rate		✓			Collection of referrals*		✓	✓
OT knowledge		✓			Knowledge questionnaire	✓	✓	✓
MG knowledge		✓			Knowledge questionnaire	✓	✓	✓
MD knowledge		✓			Knowledge questionnaire	✓	✓	✓

PO = primary outcome; SO = secondary outcome; EE = economic evaluation; BG = background; T0 = baseline measure; T1 = 6 month follow-up measure; T2 = 12 month follow-up measure; CAQ = COTiD Adherence Questionnaire; SPs = strategy providers; OTs = occupational therapists; MGs = managers; MDs = medical doctors * Referrals are collected in the one year period between T0 and T2

**Table 2:** Overview of client-caregiver outcome measures

Variable	SO	EE	BG	Instrument	T0	T1	T2	T3	T4
**Client**									
Demographics			✓	Survey	✓				
Depression			✓	GDS	✓				
Mental State			✓	MMSE	✓				
Quality of life	✓			Dqol	✓		✓		✓
Quality of life	✓	✓		EQ-5D	✓		✓		✓
Execution of daily activities	✓			AMPS	✓		✓		✓
Problems in daily life	✓			COPM	✓		✓		✓

**Caregiver**									
Demographics			✓	Survey	✓				
Quality of Life	✓			Dqol	✓		✓		✓
Quality of life	✓	✓		EQ-5D	✓		✓		✓
Clients execution of daily activities	✓			IDDD	✓		✓		✓
Sense of competence	✓			SCQ	✓		✓		✓
Problems in daily life	✓			COPM	✓		✓		✓
Depression			✓	CES-D	✓		✓		✓
Healthcare costs	✓	✓		RUD lite basic	✓				
Healthcare costs	✓	✓		RUD lite follow-up		✓	✓	✓	✓

SO = secondary outcome; EE = economic evaluation; BG = background; T0 = baseline measure; T1 = 3-month follow-up measure; T2 = 6-month follow-up measure; T3 = 9 -month follow-up measure; T4 = 12-month follow-up measure; GDS = Geriatric Depression Scale; MMSE = Mini Mental State Exam; Dqol = Dementia Quality of Life Scale; AMPS = Assessment of Motor and Processing Skills; EQ-5 D = EuroQol 5D; COPM = Canadian Occupational Performance Measure; IDDD = Interview for Deterioration of Daily Activities in Dementia; SCQ = Sense of Competence Questionnaire; CES-D = Center for Epidemiologic Studies Depression Scale; RUD Lite basic = Resource Utilisation in Dementia - Baseline questionnaire; RUD Lite follow-up = Resource Utilisation in Dementia - follow-up questionnaire

#### Primary outcome measures

Use of community OT is defined as the number of clients with dementia referred to community OT according to the COTiD program (either specific or non-specific) compared to the total number of referrals of people with dementia to community OT services. Specific referrals are those in which the name of the program is mentioned (e.g. OT according to the COTiD program). Non-specific referrals contain a referral question in which the physicians requests evaluation, therapy, or advice concerning daily activities in the home environment of the client and/or caregiver. Referrals concerning only advice regarding an aid (singular questions) are only included in the total number of referrals collected.

Data on referrals are collected by requesting participating OTs from each cluster to send copies of all referrals concerning community OT for people with dementia and/or their caregiver to the research team.

Adherence of OTs to the COTiD program is defined as 'the degree to which OTs intent to treat clients with dementia and their caregivers according to the COTiD program'. The use of Standardized Patients (SP) can be seen as the golden standard to measure adherence [17], however, this is a costly method. Closed ended questionnaires are commonly used to gather data, but are likely to evoke socially desirable answers. In addition, respondents tend to overestimate their behavior. Therefore, we will use vignettes, which seems a more valid method compared to questionnaires and more feasible than SPs. Vignettes are simulations of realistic events used to obtain participants' knowledge, attitudes, or opinions on how they would behave in a theoretical situation [18]. Previous studies [17,19] showed that vignettes provide sufficiently valid data to measure adherence. In addition, they were found to be sensitive to variation in setting [17] and suitable for creating a sufficient case-mix [17,20].

Two vignettes were created and reviewed by an expert panel. Open-ended questions are used to avoid overestimation of adherence due to cues in the questions [21]. The same questions were used for both vignettes. All questions are based on quality indicators that are based on the COTiD program and defined by experts and consensus rounds of OT's (Döpp, van 't Leven, Kaijen, de Swart, Vernooij-Dassen, Graff: Quality Indicators for Community Occupational Therapy for People with Dementia and their Caregivers: Development and Testing, in preparation).

In order to evaluate change in adherence over time and change between research-groups, data gained through the vignettes are quantified using a standardized scoring system. This system will assist in producing an adherence percentage between 0% (no adherence) and 100% (complete adherence). The content of the scoring system is based on quality indicators and was reviewed by an expert panel. Inter-rater reliability of the scoring system will be evaluated prior to data analysis.

As the use of vignettes is fairly new an additional close-ended questionnaire is developed to gather data on adherence to the COTiD program. The questionnaire contains questions on the frequency OTs perform different activities. OTs are asked to rate the frequency of these activities on a five-point scale from "never" to "always". Data gathered through this questionnaire as well as data provided by the web-based system will be used for validation purposes.

Both the vignettes and the close-ended questionnaire were formatted in an electronic survey system. Participants were provided with a personal link through e-mail to get access to these questionnaires.

#### Secondary outcome measures on professional level

Healthcare professionals' knowledge about the COTiD program is measured using a close-ended electronic questionnaire. The focus of each questionnaire is adapted to the knowledge required for each group of professionals (OTs, managers, and physicians). The questionnaires were evaluated by an expert panel.

#### Secondary outcome measures on client-caregiver level

To evaluate the effect of the CI-strategy on client-caregiver couples, treatment outcomes are measured. Table 2 shows which information is collected. Demographic information collected concerns age, marital status, education, (previous) profession, disease, disabilities, and relationship between client and caregiver. Data collection takes place at the client and/or caregivers home environment.

### Sample size and power calculations

We developed a cluster randomized trial, with randomization at institute level. Adherence of OTs to the COTiD program and the use of community OT are both primary endpoints. We hypothesize that the experimental intervention will increase OTs' adherence to the COTiD program from 20% to 50% and increase the use of community OT from 5% to 25%. Per institute, two OTs are included and on average at least 10 clients are expected to be eligible for community OT. The Intra cluster Correlation Coefficient (ICC) of OT within institutes (with respect to adherence) and the ICC of clients within institutes (with respect to OT use) is assumed to be 0.05. Corrected for the clustering of OTs within institutes, the 'effective' sample size of each cluster (institute) with respect to adherence is 1.9 OTs (= n/[1+(n-1)*ICC], where n is the number of OTs per institute (i.e. 2) and ICC is the intra cluster correlation of OTs within an institute (i.e. 0.05)). Corrected for clustering of clients within institutes, the effective sample size of clusters with respect to community OT use is 6.8 clients (n = 10, ICC = 0.05). Therefore, randomizing 30 clusters to control and 15 to intervention provides the same power for adherence as an individually randomized trial of 57 (= 30 × 1.9) subjects on control versus 29 (= 15 × 1.9) subjects on intervention, where the subjects are independent (not correlated within clusters). Thus, this cluster randomized trial provides 80% power to detect an increase from 20% to 50% in adherence. Similarly, this trial provides the same power for use of community OT as an individually randomized trial of 204 (= 30 × 6.8) subjects on control and 102 subjects on intervention. Thus, this cluster randomized trial provides 99% power to detect an increase from 5% to 25% in community OT use. The combined power for both endpoints then is at least 0.8*0.99 = 79% [22].

### Informed consent and ethical approval

In the Netherlands studies involving human subjects need to undergo a medical ethics review if they are subject to the Medical Research Involving Human Subjects Act (WMO). Studies involving completing questionnaires do not generally bring a study within the scope of this Act. To be sure the research team did submit materials to the Human Subjects Committee of the region Nijmegen/Arnhem. This committee decided that the questionnaires in our study were not too burdensome for participants including the people with dementia and their caregivers. Therefore, the study was exempt from further review by the Human Subjects Committee.

All participants were requested to sign a consent form prior to data collection. Professionals and client-caregiver couples are participating voluntarily and can stop participation at any time.

### Statistical methods

Random effects regression models will be used to evaluate differences in adherence and in use of community OT between the experimental and control group. Baseline scores will be used as covariates and type of setting and OT will be used as random factors.

Differences in knowledge between professionals (occupational therapists, physicians, and managers) in the experimental and control group will be evaluated using t-tests, unless data have a substantially skew distribution in which case non-parametric tests are used. Random effect regression models for repeated measures will be used to evaluate differences in knowledge at different times of measurement within each group and between groups. The influence of several characteristics of the professionals on their knowledge level will be evaluated using ANOVA (e.g. sex) and linear regression (e.g. age, years of professional experience).

Random effects regression models will be used for analyses of covariance of the outcome measures (AMPS process [23], IDDD performance [24], DQOL [25], SCQ [26], EQ-5 D [27] at 6 and 12 months (see Table 2)) based on an intention-to-treat analysis of all available data. Treatment differences between baseline and 6 months and baseline and 12 months will be computed by analysis of covariance, with age, sex, relation to patient, and baseline scores on the co-morbidity, MMSE scores, GDS scores, and outcome variable at baseline as covariates.

For all tests significance will be tested using two-sided tests with an alpha level of.05.

### Economic evaluation

One of the primary questions of this study concerns the difference in cost-effectiveness between the CI-strategy and the ED-strategy strategy regarding adherence of OTs to the COTiD program. Secondary, the study is designed to evaluate the difference in cost-effectiveness between the implementation strategies with regard to the quality of life of clients with dementia and their caregiver. To evaluate these questions an economic evaluation will be executed from a societal viewpoint. This implies that both costs within and outside the healthcare system, are included in the evaluations [28,29].

#### Costs

Table 3 displays data collected on costs of the implementation strategies. All costs made for the execution and development of the two strategies are registered. Developmental costs are calculated using the annuitization procedure [28]. Because of the unbalanced design (more clusters are randomly assigned to the control group) the calculation of costs will not be protocol driven. This prevents differences in costs between groups due to an unequal number of OTs, physicians, managers and/or clients.

**Table 3:** Cost data collected on the implementation strategies

Costs/Time	T0	T1	T2
**Occupational Therapists (OTs)**		✓	
Time spend on post-graduate course		✓	
Time spend on the implementation training		✓	
Time spend on coaching on the job		✓	✓
Time spend on regional meetings		✓	✓
			
**Physicians**			
Time spend on reading newsletters		✓	✓
Time spend on reading on the website		✓	✓
Time spend on motivational phone calls		✓	✓
			
**Managers**			
Time spend on reading newsletters		✓	✓
Time spend on reading on the website		✓	✓
Time spend on motivational phone calls		✓	✓
			
Development & execution*			
Post-graduate course - developmental & execution costs			
Implementation training - developmental & execution costs			
Regional meetings - developmental & execution costs			
Coaching on the job - developmental & execution costs			
Web-based system - developmental costs			
COTiD-program website - developmental costs			
Newsletters - preparation costs			
Motivational phone calls - preparation & execution costs			

T0 = baseline measure; T1 = 6 month follow-up measure; T2 = 12 month follow-up measure/* All development and execution costs are registered throughout the entire study period.

Healthcare costs made by client-caregiver couples are collected using the Lite version of the resource utilization in dementia instrument (RUD Lite) [30]. The RUD Lite is used every 3 months during a one year period. Data on both the caregiver and client are provided by the informal caregiver. Caregivers are asked to provide information about the preceding month as retrieving information over a longer period is often unreliable. An algorithm will be used to get a reliable estimate for the total period of three months. If available, market prices are used to calculate costs. If these are not available standard cost-prices are used as identified in the Dutch manual for costs in economic evaluations [31].

#### Cost-effectiveness and cost-utility analysis

In order to evaluate the cost-effectiveness of the two implementation strategies regarding OT adherence to the COTiD program, incremental cost-effectiveness ratios are determined expressed as cost per extra percentage adherence.

The cost-utility with regard to the treatment effects of the two implementation strategies is evaluated by determining incremental cost-utility ratios. These are expressed as cost per patient quality adjusted life year (QALY) gained and cost per caregiver QALY gained. QALYs are calculated using the scores on the EQ-5 D [27]. The EQ-5 D scores are converted to QALYs using the EQ-5 D health tariffs for the Dutch population [32].

For both analyses parametric uncertainty is handled by presenting acceptability curves resulting from bootstrap replications on the original sample. Deterministic uncertainty is covered by sensitivity analyses on the range of extremes of uncertain parameters [28].

### Process evaluation

The process evaluation is executed to explain the success or failure of the CI-strategy. In order to answer this overall question we evaluate (1) the exposure of healthcare professionals to the CI-strategy and (2) identify factors for success and failure of the CI-strategy as identified by the healthcare professionals.

#### Exposure to the CI-strategy

Actual exposure of healthcare professionals to the CI-strategy is evaluated using a variety of methods. Attendance and exposure to all parts of the CI-intervention is registered for each healthcare professional. The characteristics of all interventions (e.g. frequencies, duration, medium, content etc.) were registered on recording forms. The research team has unlimited access to the web-based-system and discussion platform to collect data on the frequencies these systems are used. Exposure to the website and newsletters was evaluated using a close-ended questionnaire. This questionnaire addresses the frequency participants visited the educational website and the number of newsletters read.

Actions undertaken by the research team regarding both the CI-strategy and the research process are registered in a research log.

#### Factors of success and failure of the CI-strategy

Factors for success and failure as experienced by the healthcare professionals will be identified using qualitative methods. OTs are asked to participate in a focus group discussion. Two focus groups are organized with each between 8 and 12 participants. All OTs from the experimental group will be requested to participate to make sure there are enough OTs participating in the focus groups.

Ten managers and 15 physicians will be asked to participate in a telephone interview.

Participants will be selected using purposive sampling to create a balanced mixture of professionals based on setting, age, knowledge, and referral rates. A topic list will be used to guide the focus groups and interviews. Both will be audio taped (after consent) and written out verbatim. The data are analyzed with Atlas.ti [33].

## Discussion

### Strengths

Implementation research is fairly new in the field of occupational therapy, making this a unique study. The strength of this implementation study is that not only a thorough evaluation is executed on the effects of the CI-strategy on professional practice, but that the effects on client and caregiver treatment outcomes are evaluated as well. Creating change in client-caregiver outcomes is most important as change solely on a professional level does not improve healthcare.

### Limitations

The CI-strategy is a multifaceted strategy as previous literature shows that combining two or more strategies is most effective. Because more than one strategy is used the results of this study will only show the effect of the entire package of strategies offered. We will not be able to tell in a quantitative way which strategy was more effective than another strategy. We do try to evaluate this using interviews and focus group discussion with participating professionals.

## Competing interests

The authors declare that they have no competing interests.

## Authors' contributions

MVD (scientific oversight) and MG (principle investigator), designed the study and acquired funding for the study. The research protocol was refined by CME (researcher and PhD student). During this process she was advised by MVD, MG, MO, RNS en ST. CME wrote the first draft of the manuscript, and was responsible for revisions. All authors read and approved the final manuscript.

## Pre-publication history

The pre-publication history for this paper can be accessed here:

http://www.biomedcentral.com/1471-2318/11/13/prepub
